# Preparation and Characterization of Bioplastics Based on Sweet Potato Peel Starch, *Aloe vera* and Eucalyptus Oil

**DOI:** 10.3390/polym17212950

**Published:** 2025-11-05

**Authors:** Mercedes Puca-Pacheco, María Guadalupe Neira-Velázquez, Gonzalo Canché-Escamilla, Melanie Ascue-Caballero, Alvaro Adrian Girao-Sánchez, César Augusto Tacuri-Puca

**Affiliations:** 1 Facultad de Química e Ingeniería Química, Universidad Nacional Mayor de San Marcos, Lima 15081, Peru; melanie.ascue@unmsm.edu.pe (M.A.-C.); alvaro.girao@unmsm.edu.pe (A.A.G.-S.); 2 Centro de Investigación en Química Aplicada (CIQA), Blvd. Enrique Hermosillo No. 140, Coahuila 25294, Mexico; guadalupe.neira@ciqa.edu.mx; 3 Centro de Investigación Científica de Yucatán A.C., Unidad de Materiales, Yucatán 97205, Mexico; gcanche@cicy.mx; 4 Facultad de Ingeniería Industrial, Universidad Nacional Mayor de San Marcos, Lima 15081, Peru; cesar.tacuri@unmsm.edu.pe

**Keywords:** bioplastic, sweet potato starch, eucalyptus oil, *Aloe vera*

## Abstract

The aim of this study was to produce and characterize bioplastics derived from sweet potato peel starch, *Aloe vera* gel, and eucalyptus essential oil. Starch from sweet potato peels was extracted using a wet method, yielding 3.54%, while eucalyptus oil was obtained via steam distillation, with a yield of 1.4%. In order to assess the influence of *Aloe vera* and eucalyptus oil concentrations on the properties of bioplastics, a 2^2 factorial design was implemented. Consequently, bioplastic films were produced using the casting technique. As a result, the films appeared brown, translucent, and homogeneous, while also exhibiting a rough surface texture. Mechanical testing revealed that the films possessed a high Young’s modulus of 41.1 ± 11.1 MPa, a maximum tensile strength of 2.1 ± 0.4 MPa, and an elongation at break of 21.6 ± 4.3%. These properties were achieved with a formulation containing 70% *w*/*w Aloe vera*, 0.6% *w*/*w* eucalyptus oil, and 5% *w*/*w* sweet potato peel starch, suggesting a promising eco-friendly alternative to conventional plastics for potential use in packaging applications.

## 1. Introduction

Sweet potato (*Ipomoea batatas*) is cultivated across more than one hundred countries worldwide, with China being the largest producer, contributing approximately 54.07% of global output in 2022. It is recognized as the fifth most significant food crop in developing nations due to its adaptability and nutritional importance [1,2,3]. In Peru, its cultivation spans a wide range of ecological zones—from lowland coastal valleys to the high jungle and inter-Andean valleys situated between 500 and 2000 m above sea level [4,5].

Globally, sweet potato exists in numerous varieties, including white, purple, pink, orange, and yellow types, among which the yellow and purple varieties are the most commercially prominent. During both domestic and industrial processing, substantial quantities of peel waste are generated, as the tubers are generally peeled prior to consumption or transformation. This by-product is commonly discarded, contributing to environmental pollution; however, valorizing this agricultural residue could offer both ecological and economic advantages [6,7].

The peels of sweet potatoes are rich in water, carbohydrates, dietary fiber, vitamins, minerals, and various bioactive compounds [6], making them a suitable raw material for bioplastic production. Bioplastics currently represent about 1% of the more than 360 million tons of plastics produced globally each year [8].

Bioplastics are polymeric materials obtained wholly or partially from renewable feedstocks such as starch, cellulose, plant-derived oils, or microbial biomass. Their classification is primarily determined by two main factors: the origin of the raw materials (bio-based or fossil-based) and their biodegradability. According to these criteria, bioplastics can be grouped into three major categories: (a) bio-based and biodegradable polymers—for instance, polylactic acid (PLA), polyhydroxyalkanoates (PHA), and thermoplastic starch (TPS)—which are synthesized from renewable sources and can undergo degradation under suitable environmental conditions; (b) bio-based but non-biodegradable plastics, such as bio-polyethylene (bio-PE) and bio-polyethylene terephthalate (bio-PET), which are derived from biological resources but remain resistant to natural decomposition; and (c) fossil-based biodegradable plastics, including polybutylene adipate terephthalate (PBAT), which are produced from petrochemical precursors yet retain the capacity to biodegrade [9].

Understanding these distinctions is crucial when evaluating the environmental footprint and end-of-life management of plastic alternatives [10]. Despite their advantages, bioplastics often face limitations, including low mechanical strength, high production costs, and insufficient recycling or composting infrastructure. Many of them require industrial composting conditions to fully degrade, creating challenges for large-scale implementation within a circular economy [11]. Nevertheless, bioplastics are increasingly applied in packaging, agriculture, medicine, and textiles, offering eco-friendly substitutes for conventional plastics [12].

Among biodegradable options, starch-based bioplastics are particularly attractive for mitigating pollution caused by petroleum-derived plastics. However, pure starch films typically lack the mechanical robustness and barrier properties required for industrial use [13]. To improve these characteristics, various reinforcing agents or additives—such as natural fibers, cellulose nanostructures, metal oxide nanoparticles, and essential oils—are incorporated into starch matrices [14].

The conversion of agricultural residues into bioplastics represents a sustainable and environmentally responsible strategy, aligning with circular economy principles. Agricultural by-products like husks, stalks, sugarcane bagasse, and corn residues offer renewable and inexpensive feedstocks while reducing dependence on petroleum-based polymers. Utilizing these organic wastes for bioplastic synthesis helps mitigate soil and aquatic pollution by minimizing the accumulation of conventional plastics. Huilcarema-Enríquez et al. (2023) emphasized that this approach not only reduces environmental impacts but also promotes technological and socioeconomic development in rural agricultural regions [15].

Nature provides numerous bio-based compounds that can enhance the performance of biodegradable materials. *Aloe vera* gel, extracted from the leaves of *Aloe barbadensis* Miller, contains a high concentration of mucilage stored in the mesophyll, serving as a natural water reservoir. It is rich in carbohydrates and phenolic compounds, which provide antioxidant properties [16]. Meanwhile, essential oils are complex mixtures of terpenes, terpenoids, and various aromatic and aliphatic compounds, characterized by low molecular weights and distinctive odors [17,18,19,20]. Eucalyptus essential oil, in particular, functions as a natural plasticizer, improving flexibility and toughness while exhibiting antibacterial activity [21].

The originality of this study lies in the development of biodegradable bioplastic films produced from sweet potato peel starch, *Aloe vera* gel, and eucalyptus essential oil—utilizing an agricultural waste product that is usually discarded, thereby adding value to it. *Aloe vera* contributes to improved elasticity and tensile strength, counteracting the brittleness typically observed in starch-based films. Although the antimicrobial properties of eucalyptus oil were not evident in this research, its hydrophobicity may help decrease water vapor permeability.

In summary, the synergistic combination of these natural ingredients enhances the overall performance of the resulting bioplastic and provides an eco-friendly alternative to fossil-based materials. This work contributes to advancing the development of sustainable and biodegradable packaging solutions.

## 2. Materials and Methods

### 2.1. Materials

Yellow sweet potato tubers (*Ipomoea batatas*) and *Aloe vera* leaves (*Aloe barbadensis* Miller) were collected in May 2023 from the Lima region of Peru. Glycerin (Alkofarma E.I.R.L., Lima, Perú), distilled and deionized water were utilized in the formulation of bioplastics.

### 2.2. Methods

#### 2.2.1. Extraction of Starch from Yellow Sweet Potato Peels

Starch was isolated from the peels of yellow sweet potatoes through a wet extraction technique. Initially, the tubers were harvested, sorted, and thoroughly rinsed with clean water. The outer skins were manually removed from the flesh and cut into small fragments. These fragments were homogenized using an industrial-grade blender, maintaining a peel-to-deionized water mass ratio of 1:2. The resulting blend was passed through a filtration process to eliminate fibrous material, producing a slurry enriched in starch. This slurry was left undisturbed to allow sedimentation, after which the settled starch was collected and subjected to drying at 50 °C for a duration of 15 h. Following dehydration, the starch was milled and sieved using a 100-mesh screen, then stored at ambient temperature until further application. The efficiency of the extraction process was determined using Equation (1) [22]

(1) $$Starch\;yield\;(\%)=\frac{Weight\;of\;sweet\;potato\;peel\;starch\;(g)}{Weight\;of\;sweet\;potato\;peel\;(g)}\times 100$$

#### 2.2.2. Extraction of *Aloe vera* Gel

Fresh *Aloe vera* leaves were harvested and carefully washed to remove any surface contaminants. They were then immersed in potable water for 48 h to eliminate the yellow exudate containing phenolic compounds. Following this step, the outer rind was peeled off, leaving only the mucilaginous inner portion. The obtained pulp was cut into small pieces and homogenized using a blender for approximately 5 min to produce a uniform gel, which was subsequently refrigerated until further use [23].

#### 2.2.3. Extraction of Eucalyptus Oil

Fresh young eucalyptus leaves were selected, manually defoliated from branches, and weighed, showing an average moisture content of 60.95 ± 1.37%. The essential oil was extracted by steam distillation using an extraction chamber, producing a 1.4% yield based on the initial leaf mass [24].

#### 2.2.4. Preparation of Bioplastic Films

Bioplastic films were fabricated through a casting technique. Starch extracted from sweet potato peels was dispersed in distilled water according to the designed formulation and preheated to 40 °C. Subsequently, *Aloe vera* gel was incorporated and mixed at 600 rpm for 15 min. Glycerin, functioning as the plasticizing agent, was then introduced and stirred for an additional 10 min. Afterward, eucalyptus oil was added in amounts specified by the experimental plan. The resulting suspension was heated to 60–70 °C until it reached a viscous state and was then poured into 20 × 20 cm glass plates. Drying was carried out in a hot-air oven at 60 °C for 10 h, followed by cooling at ambient temperature. The films were removed from the molds after 24 h and subsequently analyzed for their physicochemical and mechanical properties.

#### 2.2.5. Experimental Design

A 2^2 factorial design was employed to evaluate the effects of two independent variables on bioplastic formulation: *Aloe vera* gel concentration and eucalyptus essential oil concentration. Each factor was tested at two levels: high (+) and low (−). All formulations included a fixed amount of sweet potato peel starch (5 g) and glycerin (2 g), the latter serving as a plasticizer, as detailed in Table 1.

#### 2.2.6. Characterization of the Components and Bioplastic Films

Fourier Transform Infrared (FTIR) spectroscopy was used to analyze the chemical structure of the raw materials and bioplastic films. Infrared spectra were recorded using a Nicolet model protege 8700 equipment with an ATR accessory featuring a ZnSe crystal. Each sample was scanned 30 times over a spectral range of 4000 to 650 cm^−1^.

Mechanical properties of the films were determined with a Shimadzu AGS-X universal tester (100 N load cell) at a crosshead speed of 3 mm/min, following the procedure described in ASTM D882-18, Standard Test Method for Tensile Properties of Thin Plastic Sheeting (ASTM International, West Conshohocken, PA, USA, 2018).

Thermogravimetric analysis (TGA) was performed to assess the thermal stability and decomposition behavior of the bioplastic films. Samples (~10 mg) were heated from 25 to 700 °C at 10 °C/min under nitrogen using a TA Instruments Q500. Mass loss was recorded continuously.

Thermal transitions of the bioplastic films were evaluated using a differential scanning calorimeter (DSC), model Q200 from TA Instruments. Approximately 5–10 mg of each sample was heated from 0 °C to 380 °C at a rate of 10 °C per minute under a nitrogen flow.

Dynamic mechanical analysis (DMA) was performed using a Perkin Elmer DMA-7 instrument. Film specimens were prepared with dimensions of 3 mm in width and 15 mm in length. The samples were heated from −100 °C to 100 °C at a rate of 5 °C/min under controlled conditions.

Surface morphology of starch and bioplastic films was observed with a JEOL 6360LV scanning electron microscope (SEM) operating at 8 kV. Before analysis, specimens were sputter-coated with a gold–palladium layer to improve image resolution, and micrographs were captured under low vacuum at 2000× magnification.

Biodegradability was assessed through aerobic degradation, which leads to complete oxidation of the bioplastic material. The gravimetric method was used to quantify mass loss. Bioplastic films (2 cm × 4 cm) were weighed in duplicate and buried 3 cm below the surface in agricultural soil contained within 1-L containers. The containers were maintained under controlled conditions of 55% relative humidity and 25 °C. Each container was hermetically sealed with Parafilm to allow microbial activity to proceed naturally, resulting in decomposition into carbon dioxide, water and/or methane, and biomass. The bioplastic films were removed and weighed at the end of the evaluation month. The standardized biodegradability test was conducted over a period of one month, using Equation (2) for the calculation.

(2) $$\%Weight\;loss=\frac{\left[Initial\;dry\;weight\;of\;the\;bioplastic\;(g)-Final\;dry\;weight\;of\;the\;bioplastic\;(g)\right]}{Initial\;dry\;weight\;of\;the\;bioplastic\;(g)}\times 100$$

CO_2_ emissions were determined using the alkaline absorption method. The tests were adapted from the principles established in ASTM D5338, Standard Test Method for Determining Aerobic Biodegradation of Plastic Materials Under Controlled Composting Conditions (ASTM International, West Conshohocken, PA, USA, 2015). In this study, agricultural soil was used in place of compost to evaluate the biodegradation of the bioplastic by measuring CO_2_ evolution under controlled conditions.

**Figure 1:**
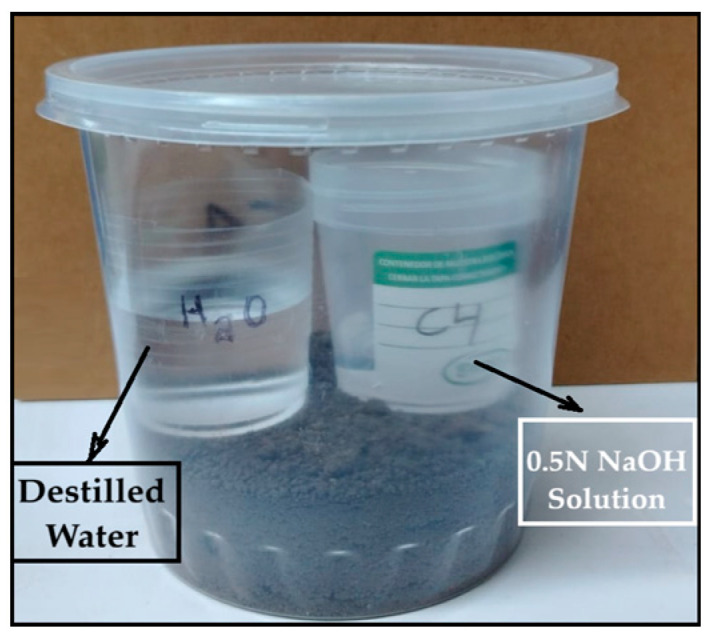
Determination of CO_2_ emissions (kg)/kg of bioplastic by the alkaline absorption method.

The test involved placing a 2 cm × 4 cm bioplastic sample—previously weighed in duplicate—into agricultural soil at a depth of 3 cm within a 1-L polyethylene container. Inside the container, two small beakers were placed: one containing 20 mL of 0.5 N sodium hydroxide (NaOH) solution to absorb CO_2_, and another with 50 mL of distilled water to maintain humidity, as shown in Figure 1.

The container was hermetically sealed with Parafilm. This procedure was repeated for all samples, including a blank control (without bioplastic). The containers were stored in a dark environment or covered with paper for periods of 7, 10, 14, 22 and 30 days. During this period, microbial activity in the soil led to the aerobic degradation of the bioplastic, releasing CO_2_, which was absorbed by the NaOH solution according to the following reaction:

CO_2_ (g) + 2 NaOH (aq) → Na_2_CO_3_ (aq) + H_2_O (aq) (3)

After one month, the NaOH solution was titrated with 0.25 N hydrochloric acid (HCl) to determine the amount of CO_2_ absorbed, based on the reaction:

NaOH (aq) + HCl (aq) → NaCl (aq) + H_2_O (aq) (4)

Using back titration, the number of moles of NaOH that reacted with CO_2_ was calculated, and subsequently converted into mass (kg of CO_2_). Separately, the bioplastic films were reweighed at the end of the test period to determine the amount of material degraded (initial mass minus final mass). The final result was expressed as kg CO_2_ emitted per kg of degraded bioplastic.

#### 2.2.7. Statistical Analysis

For the ANOVA analysis of the results, Minitab Statistical Software Version 18 (Minitab, LLC, State College, PA, USA) was used.

## 3. Results and Discussion

### 3.1. Physicochemical Characterization of Sweet Potato Peel Starch

Table 2 presents the yield and physicochemical properties of starch extracted from yellow sweet potato peels. A starch yield of 3.54 ± 0.11% was obtained from the peels analyzed in this study. The quantity of peel—and consequently the amount of starch recovered—depends on the peeling method employed. Previous studies have reported variability in peel yield depending on the technique used.

The extracted starch exhibited characteristics comparable to conventional potato starch, with an amylose content of 17.6% and an amylopectin content of 82.4%. The gelatinization temperature was 68.9 °C.

The extraction yield of *Aloe vera* gel was 60.24%, with a water content of 99.11%. In addition to water, *Aloe vera* gel contains carbohydrates, organic acids, and salts [28,29,30,31]. The carbohydrate profile includes galactose, glucose, xylose, mannose, arabinose, aldopentose, glucomannan, fructose, acemannan, pectic substances, and L-rhamnose [32,33,34].

### 3.2. Chemical Structural Characterization of Film Components and Bioplastic

The infrared (IR) spectrum of sweet potato peel starch displays a broad absorption band around 3300 cm^−1^, attributed to O–H stretching vibrations, indicating the presence of hydroxyl groups (–OH) in the starch structure. The band near 2900 cm^−1^ corresponds to C–H stretching vibrations, suggesting the presence of aliphatic groups. Bands in the 1200–900 cm^−1^ region are characteristic of C–O and C–C stretching, as well as C–H bending vibrations, typical of polysaccharides such as starch.

**Figure 2:**
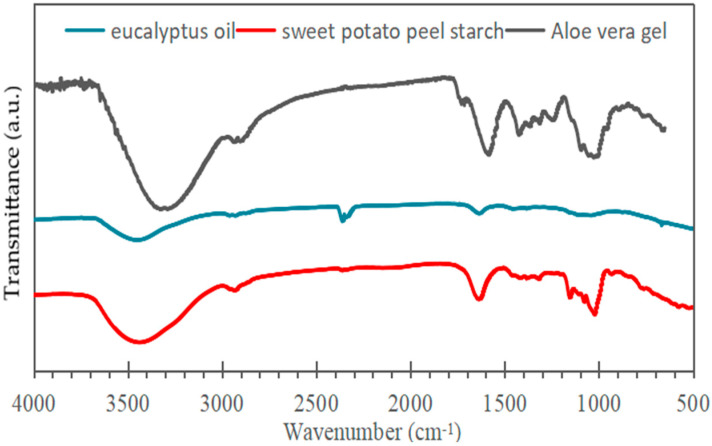
Infrared spectra of samples of aloe vera, eucalyptus oil, and sweet potato peel starch.

The FTIR spectrum of eucalyptus essential oil (Figure 2) displays a band near 1048 cm^−1^ attributed to C–O stretching in ether groups and a strong absorption at 1710 cm^−1^ corresponding to C=O vibrations of carbonyl compounds such as aldehydes, ketones, and carboxylic acids. Additional peaks between 1200 and 1400 cm^−1^ and around 1630 cm^−1^ represent C–H bending and C=C stretching of alkenes, respectively [35].

In the dried *Aloe vera* gel spectrum, a broad O–H stretching band appears between 3200 and 3600 cm^−1^ (centered at 3308 cm^−1^), typical of carbohydrate units like mannose and uronic acid [36,37]. Absorptions from 2800 to 3000 cm^−1^ are assigned to aliphatic C–H stretching, while a distinct C=O signal at 1734 cm^−1^ indicates sugars and amino acid residues. Additional features at 1634, 1578, and 1418 cm^−1^ arise from asymmetric and symmetric stretching of carboxylate groups (–COO^−^), and the band near 870 cm^−1^ is linked to out-of-plane C–H deformation of carbohydrate structures [38].

**Figure 3:**
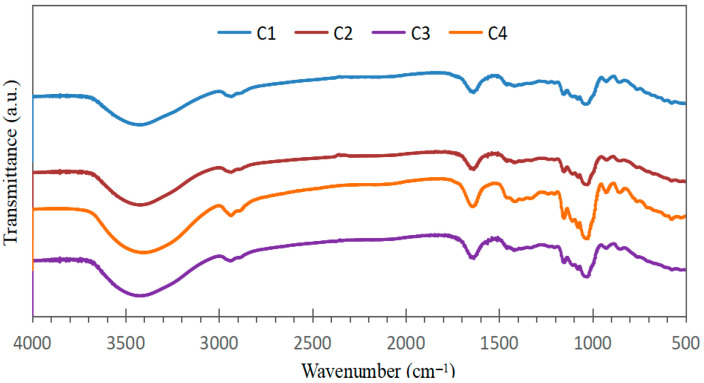
Infrared spectra of bioplastic samples at different concentrations of starch, *Aloe vera* gel and eucalyptus oil.

The FTIR spectra of the bioplastic samples (Figure 3) exhibited similar patterns, confirming the presence of common constituents derived from starch, *Aloe vera* gel, and eucalyptus oil. Variations in peak intensities likely reflect differences in the relative proportions of these components within each formulation.

### 3.3. Evaluation of the Mechanical Properties of Bioplastic Films

The produced bioplastic films exhibited a consistent and smooth structure, averaging about 150 µm in thickness. According to Table 3, the sample with greater proportions of *Aloe vera* gel and eucalyptus oil (C1) demonstrated enhanced mechanical behavior, with a Young’s modulus of 41.1 ± 11.1 MPa, maximum tensile strength of 2.1 ± 0.4 MPa, and elongation at break of 21.6 ± 4.3%. In contrast, the film containing the lowest amounts of these additives (C2) presented inferior mechanical resistance.

Table 4 shows the analysis of variance for the mechanical properties. According to this analysis, a significant effect of *Aloe vera* concentration on Young’s modulus was obtained with a *p* value of 0.03 (*p* ˂ 0.05). However, there was no significant effect of the concentration of eucalyptus oil. This behavior can be attributed to physical interactions, such as hydrogen bonds between the monosaccharides presents in *Aloe vera* gel with the amylose and amylopectin chains of the starch. These interactions could act as cross-links between these chains and result in an increase in the mechanical strength and rigidity of the films. This effect was reported by Pinzón et al. [39], who found that the addition of *Aloe vera* gel led to more rigid films for the bioplastic of banana starch-glycerol-*Aloe vera* gel.

Although the incorporation of eucalyptus oil did not result in statistically significant differences, it contributed to improved mechanical properties of the bioplastic. This enhancement is attributed to the oil’s ability to occupy intermolecular spaces between starch chains, promoting chain mobility and resulting in more flexible bioplastic films.

The biodegradable films formulated from yellow sweet potato peel starch blended with *Aloe vera* and eucalyptus oil demonstrated superior mechanical behavior compared to those produced solely from sweet potato peel starch. The latter exhibited a Young’s modulus of 17.9 ± 2.16 MPa, a tensile strength of 1.37 ± 0.15 MPa, and an elongation at break of 18.2 ± 3.04%. Nevertheless, these parameters remain considerably lower than those of conventional petroleum-based plastics, such as polyethylene.

For instance, low-density polyethylene (LDPE) typically shows a Young’s modulus of 90.22 ± 4.25 MPa, an elongation at break of 10.25 ± 0.54%, and a tensile strength of 32.07 ± 1.85 MPa. Consequently, further improvements in the mechanical resistance of bioplastics are required to achieve competitiveness with commercial plastics [40].

The mechanical parameters obtained for the starch-based films containing *Aloe vera* and eucalyptus oil are comparable to or better than those of non-reinforced starch bioplastics, particularly regarding elongation and tensile strength. In the research conducted by Abotbina et al. (2021), a corn starch-based bioplastic plasticized with natural additives exhibited a Young’s modulus between 25 and 35 MPa, a tensile strength ranging from 1.5 to 2.5 MPa, and an elongation at break of 10–18%, which are lower than the values reported in the present study [41].

### 3.4. Evaluation of Thermal Properties

#### 3.4.1. Thermogravimetric Analysis of Bioplastic Films

**Figure 4:**
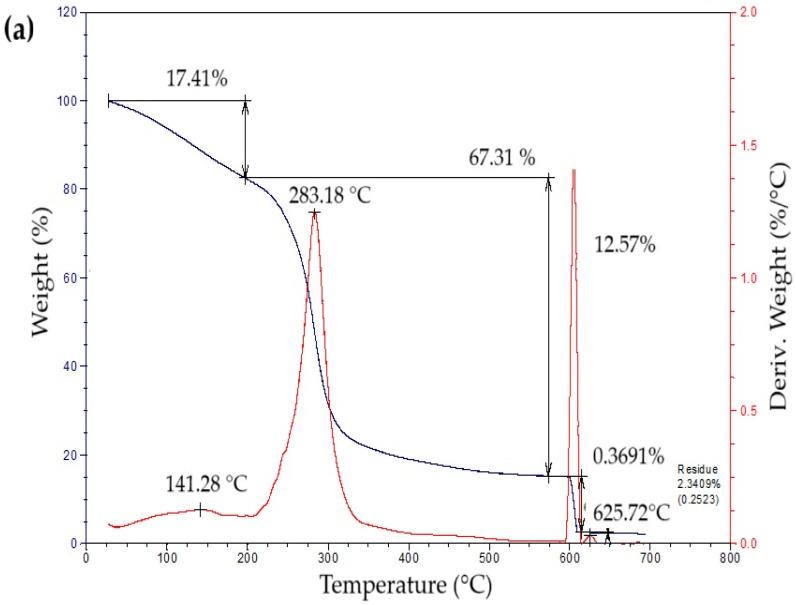

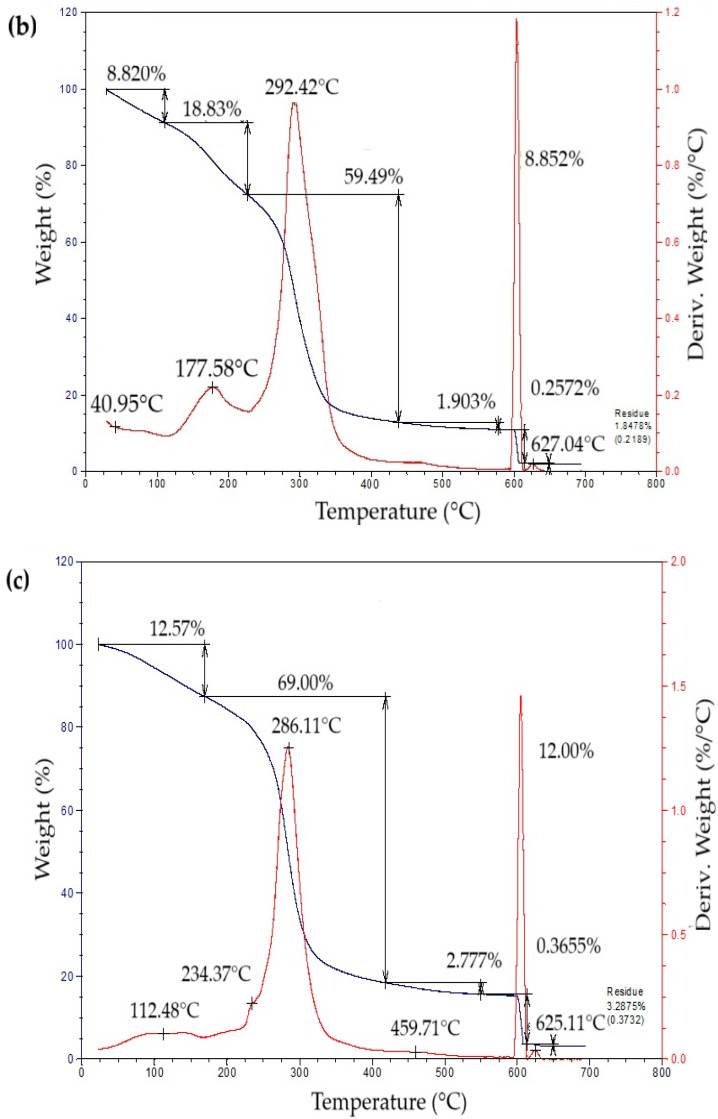

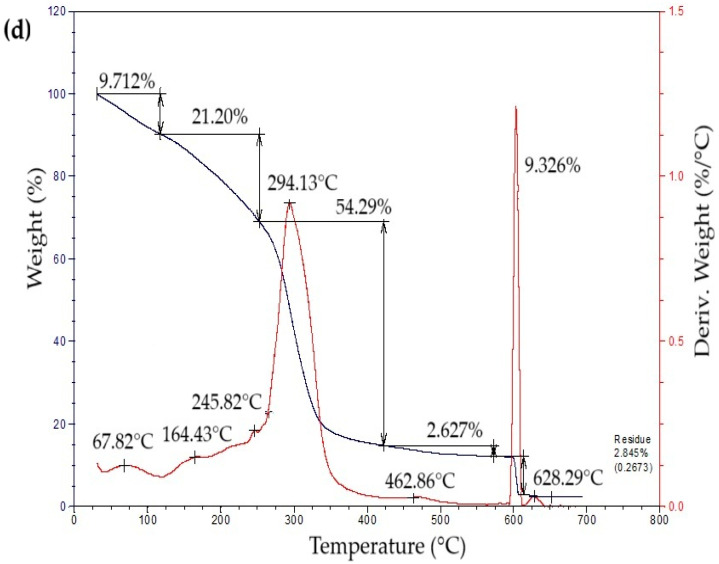
Curves of thermogravimetric analysis (TG) (black line) and differential thermogravimetric analysis (DTG) (red line) of samples of bioplastic films, such as: (**a**) C1, (**b**) C2, (**c**) C3, and (**d**) C4.

The thermogravimetric profiles of the bioplastic films (Figure 4) exhibited three sequential weight-loss stages. The initial stage, from approximately 50 °C to 260 °C, showed a gradual decrease in mass associated with water removal, volatilization of glycerol and eucalyptus oil, and thermal degradation of simple sugars. The second stage, occurring between 260 °C and 340 °C, presented a pronounced mass reduction attributed to starch decomposition [42]. This process likely involves condensation among hydroxyl groups producing ether bonds, alongside dehydration within glucose units that results in ring cleavage and the generation of unsaturated structures [43]. In the final stage, a minor additional decrease in mass was detected, leaving a residual fraction of about 15% at the end of the thermal degradation process.

Table 5 summarizes the mass loss and maximum decomposition temperature (TDmax) of the bioplastic samples. The TDmax value, determined from the peak of the derivative thermogravimetric (DTG) curve, indicates the temperature at which degradation occurs most rapidly. Films enriched with higher amounts of *Aloe vera* gel (C1 and C3) demonstrated greater thermal resistance than those with lower contents (C2 and C4). Similar behavior was reported by Kaur et al. [44], who observed enhanced thermal performance in chitosan films incorporating *Aloe vera*.

#### 3.4.2. Analysis by DSC of Bioplastic Films

**Figure 5:**
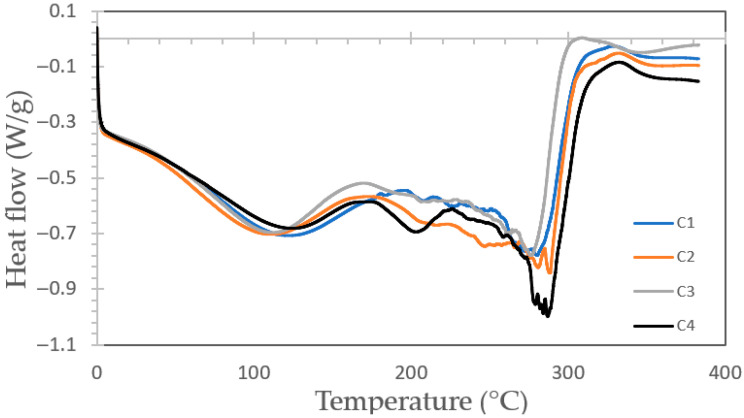
Curves of heat flow vs. temperature thermograms for bioplastic samples with formulations C1, C2, C3 and C4.

Figure 5 illustrates the Differential Scanning Calorimetry (DSC) thermograms of the bioplastic films. Two broad endothermic regions were identified, the first between 104.4 °C and 115.2 °C and the second from 276.8 °C to 288.6 °C. These signals likely correspond to the mass loss detected in the TG profiles, associated with the volatilization of small organic molecules and the decomposition of mono- and polysaccharides within the matrix. Due to the overlap of these degradation phenomena, no well-defined thermal transitions were observed across the examined temperature range.

#### 3.4.3. Dynamic Mechanical Analysis

**Figure 6:**
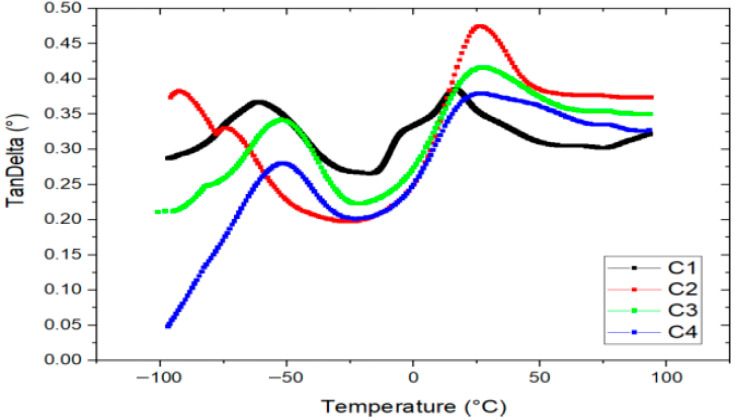
Tangent Delta vs. temperature of bioplastics with formulations C1, C2, C3 and C4.

Figure 6 presents the tan delta (tan δ) curves of the bioplastic films obtained through Dynamic Mechanical Analysis (DMA). Two distinct peaks are observed in all formulations, indicating the presence of two glass transition events associated with the plasticized starch matrix, which forms the continuous phase of the films.

The first peak, located at −52 °C, corresponds to a starch phase highly plasticized with glycerol, while the second peak, at 26 °C, is also attributed to a glycerol-rich starch phase. These transitions suggest differences in the distribution and integration of glycerin within the polymer matrix [45]. Glycerin is known to intercalate between amylose and amylopectin chains in starch, enhancing chain mobility and thereby increasing film flexibility.

Additionally, *Aloe vera* gel—rich in polysaccharides such as acemannan [46]—and eucalyptus oil may also contribute to plasticization, further improving the mechanical performance of the bioplastic films.

### 3.5. Morphological Evaluation of Bioplastic Films

**Figure 7:**
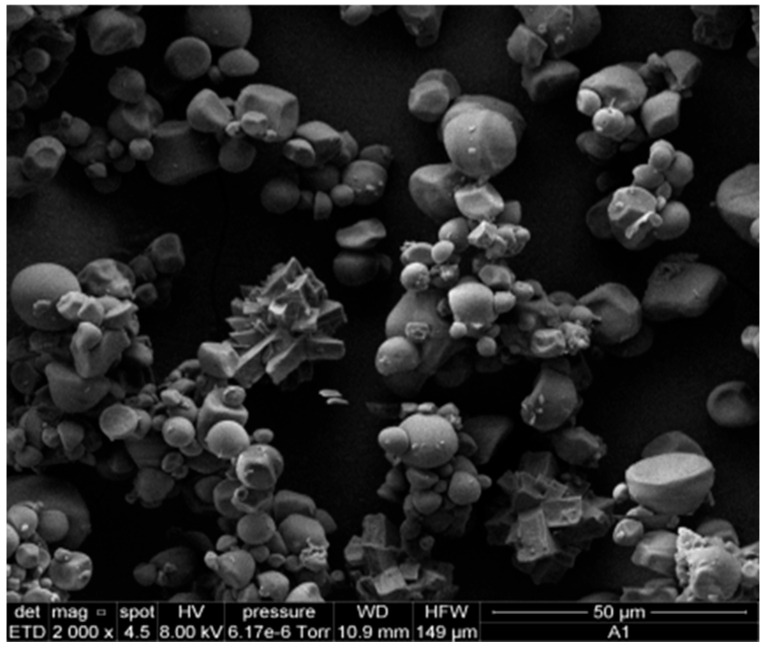
Micrograph of SEM of particles of sweet potato peel starch.

**Figure 8:**
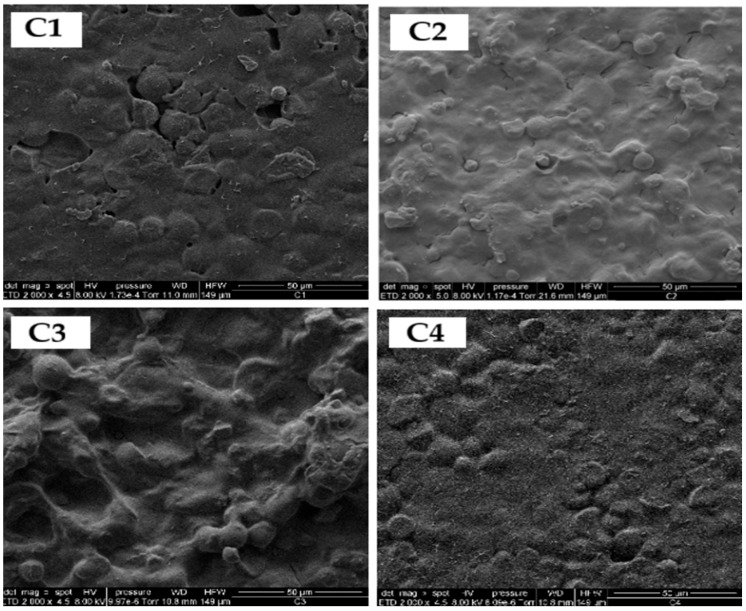
Micrographs of SEM of sweet potato peel starch-based bioplastic films (C1, C2, C3 and C4) according to Factorial design 2^˄^2.

Figure 7 shows the SEM micrograph of starch isolated from yellow sweet potato peels, revealing predominantly spherical granules with slight surface distortions. Image analysis was performed using ImageJ software (Version 1.53t, National Institutes of Health, Bethesda, MD, USA, 2023), which indicated an average particle diameter of 6.49 µm. As illustrated in Figure 8, the resulting bioplastic films exhibited a uniform structure with a moderately rough texture.

Samples containing greater amounts of eucalyptus oil (C1 and C4) displayed enhanced film integrity, likely attributed to the combined plasticizing effect of *Aloe vera* gel [47] and eucalyptus oil [48], which improves matrix flexibility and workability. However, maintaining appropriate essential oil concentrations is crucial to avoid phase separation and morphological irregularities caused by hydrophobic and density differences. Although starch gelatinization was incomplete, the granules appeared well dispersed within the polymeric network.

**Figure 9:**
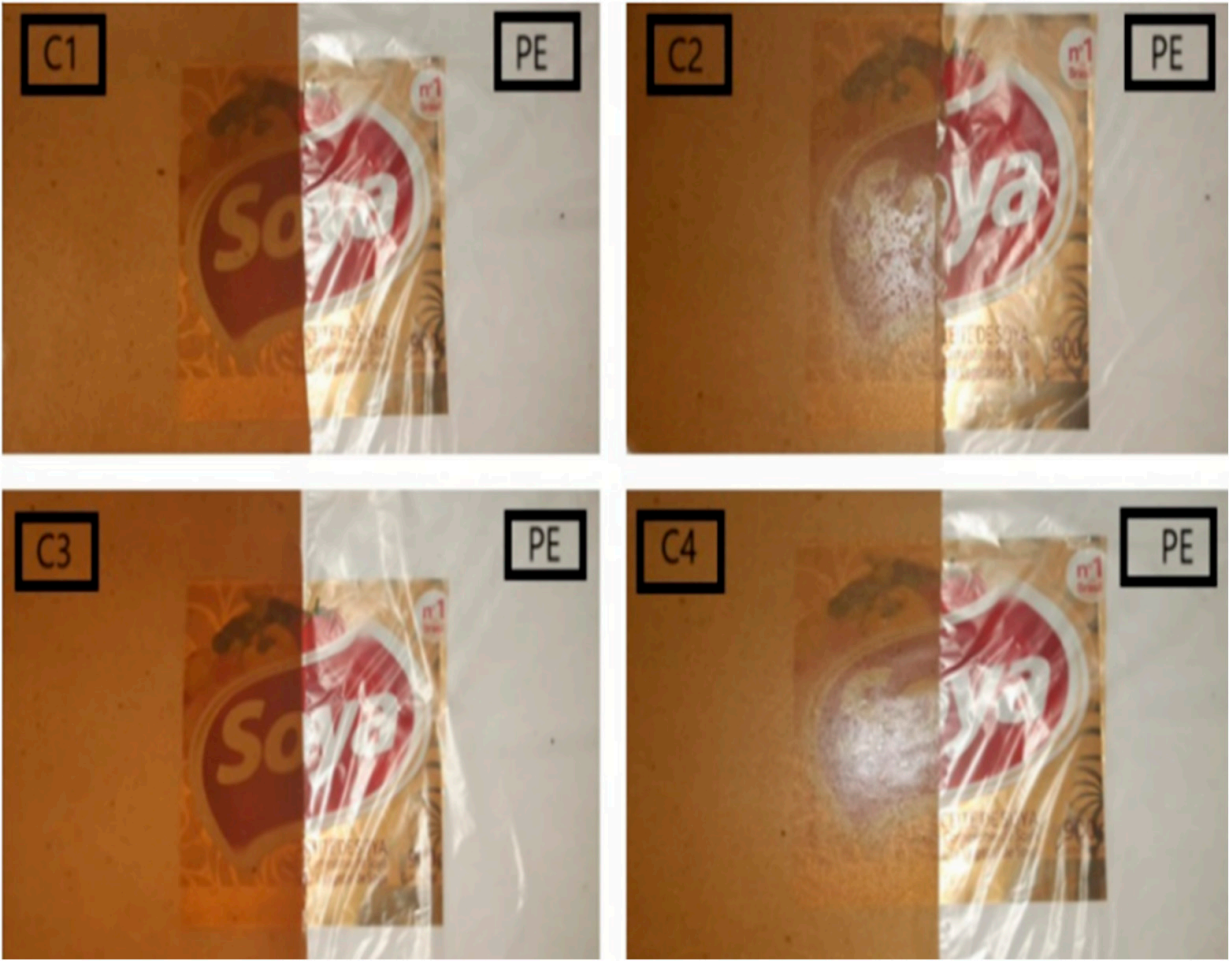
Comparative image of the transparency of bioplastic films (C1, C2, C3 and C4) with polyethylene films (PE).

Figure 9 shows the bioplastic films from formulations C1–C4, characterized by a translucent brown tone. This coloration mainly results from the presence of eucalyptus oil, whose 1,8-cineole component promotes oxidation catalyzed by polyphenol oxidase (PPO)—an enzyme retained from the sweet potato peel after starch extraction—leading to quinone formation and subsequent melanin polymerization during film development [49]. The Maillard reaction further intensifies the color through interactions between reducing sugars in starch and amino acids from *Aloe vera* gel, producing brown melanoidins. In contrast, the polyethylene (PE) control film appears colorless and transparent due to its simple polymeric structure and low light scattering.

### 3.6. Biodegradability Assessment

According to the experimental design, the biodegradation rates of the bioplastic formulations were C1 (75.08%), C2 (67.83%), C3 (60.13%), and C4 (70.89%). The degradability of these materials is mainly governed by the hydrophilic character of starch, which, in combination with glycerol, enhances water uptake and promotes microbial activity. This facilitates enzymatic cleavage of glycosidic linkages within the starch matrix [50].

Despite this, the obtained biodegradation percentages were lower than those reported by Puca et al. (2022) for starch–*Aloe vera*–graphene films (75.6–94.4%) [51]. This reduction may be linked to the antimicrobial action of eucalyptus oil and Aloe vera, which inhibits microbial colonization and thereby extends film durability. Puca et al. (2024) further demonstrated that eucalyptus oil suppresses *Staphylococcus aureus* growth and inhibits the fungi *Rhizopus stolonifer* and *Aspergillus niger* through the Kirby–Bauer diffusion assay [24]. Future research will quantify the antimicrobial activity directly within these bioplastic matrices.

Overall, the produced films present a clear environmental advantage, showing measurable biodegradability compared with conventional polymers such as polyethylene, which may persist for over a century under natural conditions, depending on exposure to light, temperature, oxygen, and microbial presence [52].

### 3.7. Evaluation of CO_2_ Emissions from Bioplastic Degradation

**Figure 10:**
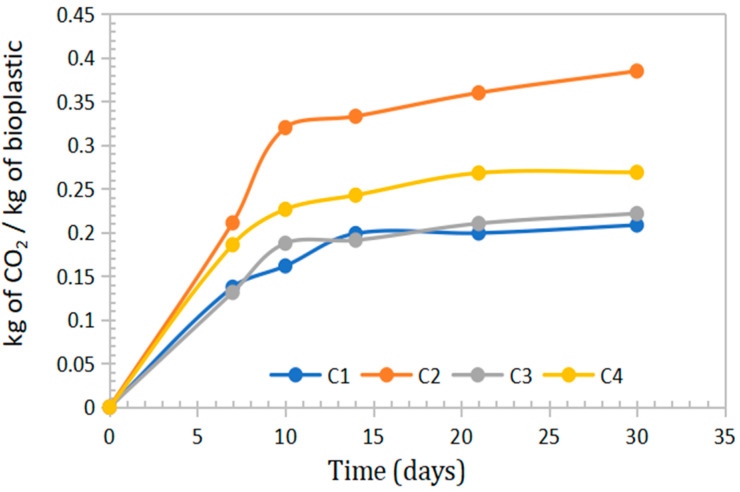
Curves on the mass emission of CO_2_ in kg generated per kg of bioplastic for periods of 7, 10, 14, 22 and 30 days of evaluation.

In the context of growing consumer awareness regarding environmental impact, it is important to note that bioplastics decompose predominantly into CO_2_ and H_2_O. Microorganisms convert the material into carbon dioxide, water, and biomass. Bioplastics derived from sweet potato peel starch, *Aloe vera* gel, and eucalyptus oil emitted between 0.2084 and 0.3847 kg of CO_2_ per kilogram of material during the degradation process, as shown in Table 6 and Figure 10.

CO_2_ emission values ranging from 0.3 to 0.6 kg CO_2_/kg have been reported during active composting for bioplastics such as PLA and PHB. In contrast, polyethylene showed negligible emissions due to its low rate of decomposition [53].

Table 7 presents the analysis of variance (ANOVA) for the mass of CO_2_ released (in kg) per kilogram of bioplastic. The results indicate a statistically significant effect of *Aloe vera* gel and eucalyptus oil concentrations, as well as their interaction, with a *p*-value < 0.005.

Based on the main effects plots, it was observed that increasing the concentrations of *Aloe vera* and eucalyptus oil led to a reduction in CO_2_ emissions. This inhibitory effect on biodegradation is primarily attributed to the antimicrobial and antifungal properties of both components, which limit microbial activity responsible for bioplastic decomposition.

## 4. Conclusions

Homogeneous, rough-textured, and biodegradable bioplastic films were successfully developed, exhibiting favorable mechanical properties. At a high concentration of *Aloe vera* gel (70% *w*/*w*) and a low concentration of eucalyptus oil (0.6% *w*/*w*), the films achieved a Young’s modulus of 41.1 ± 11.1 MPa, a maximum tensile strength of 2.1 ± 0.4 MPa, and an elongation at break of 21.6 ± 4.3%. Statistical analysis confirmed that *Aloe vera* concentration had a significant effect on Young’s modulus.

Thermogravimetric analysis revealed two distinct decomposition phases: the first, occurring between 50 °C and 260 °C, was associated with moisture loss and volatilization of glycerin and eucalyptus oil; the second, between 260 °C and 340 °C, corresponded to starch degradation.

Dynamic mechanical analysis identified two glass transition temperatures at approximately −52 °C and 26 °C.

SEM analysis confirmed spheroidal starch particles with an average diameter of 6.49 µm and their integration into the bioplastic matrix, despite incomplete gelatinization. Films with higher eucalyptus oil content showed improved flexibility and processability.

Biodegradability ranged from 60.13% to 75.08%, with lower values observed in formulations containing higher concentrations of eucalyptus oil and *Aloe vera*, due to their antimicrobial properties. CO_2_ emissions during degradation ranged from 0.2084 to 0.3847 kg CO_2_/kg of bioplastic.

These findings indicate that the synergistic incorporation of aloe vera and eucalyptus oil improves the mechanical and thermal properties of the bioplastic, while also influencing its biodegradability. This formulation represents a promising alternative to conventional petroleum-based plastics, particularly for potential use in packaging applications.

## Figures and Tables

**Table 1:** Formulations used in the experimental runs for bioplastic production, according to Factorial design 2^˄^2.

Code	Experimental Run (Aloe) (Oil)	*Aloe vera* Gel Concentration * (% *w*/*w*)	Eucalyptus Oil Concentration (% *w*/*w*)	Gliceryn (% *w*/*w*)	Starch (% *w*/*w*)	Water (% *w*/*w*)
C1	(+) (+)	70	0.6	2	5	22.4
C2	(−) (−)	20	0.2	2	5	72.8
C3	(+) (−)	70	0.2	2	5	22.8
C4	(−) (+)	20	0.6	2	5	86.4

* Aloe vera gel with 1% solids was used.

**Table 2:** Characterization of yellow sweet potato peel starch.

Characteristic	Yellow Sweet Potato Peel Starch	Method
Starch Yield (%)	3.54 ± 0.11	Gravimetric Method [22]
Humidity (%)	9.84 ± 1.23	AOAC 950.46 [25]
Total protein (%)	0.53 ± 0.05	AOAC 984.13 [25]
Fat (%)	0.56 ± 0.01	AOAC 2003.05 [25]
Crude fiber (%)	0.02 ± 0.01	AOAC 962.09 [25]
ELN * (%)	87.88 ± 0.05	AOAC 976.05 [25]
Ash (%)	1.44 ± 0.01	AOAC 942.05 [25]
Amylose (%)	17.56 ± 8.45	Hoover and Ratnayake [26]
Amylopectin (%)	82.44 ± 8.75	Hoover and Ratnayake [26]
Gelatinization temperature (%)	68.90 ± 1.85	Grace [27]

* Nitrogen-free extract.

**Table 3:** Results of Young’s modulus, maximum tensile strength and strain at break of bioplastic films.

Code	Experimental Run (Aloe) (Oil)	Young’s Module (MPa)	Maximum Tensile Strength (MPa)	Elongation at Break (%)
C1	(+) (+)	41.1 ± 11.1	2.1 ± 0.4	21.6 ± 4.3
C2	(−) (−)	20.6 ± 8.7	1.9 ± 0.2	15.4 ± 1.9
C3	(+) (−)	34.4 ± 8.8	1.8 ± 0.4	15.6 ± 4.4
C4	(−) (+)	29.2 ± 2.8	1.4 ± 0.1	12.7 ± 2.4

**Table 4:** Analysis of variance of mechanical properties of bioplastic films.

Source	MC Ajust.	F Value	*p* Value
Young’s modulus (MPa):			
A: *Aloe vera* concentration (% *w*/*w*)	495.368	6.99	0.030 *
B: Eucalyptus oil concentration (% *w*/*w*)	175.568	2.48	0.154
AB	2.708	0.04	0.850
Maximum tensile strength (MPa):			
A: *Aloe vera* concentration (% *w*/*w*)	0.26108	2.94	0.125
B: Eucalyptus oil concentration (% *w*/*w*)	0.01687	0.19	0.675
AB	0.51668	5.81	0.043 *
Elongation at break (%):			
A: *Aloe vera* concentration (% *w*/*w*)	62.108	62.108	0.050
B: Eucalyptus oil concentration (% *w*/*w*)	8.168	8.168	0.428
AB	56.768	56.768	0.059

* Significance level (*p* ˂ 0.05).

**Table 5:** Thermogravimetric analysis of bioplastic films.

Code	Design	First Decomposition Zone (60–260 °C)		Second Decomposition Zone (260–340 °C)	
		Loss Mass (%)	TDmax (°C)	Loss Mass (%)	TDmax (°C)
C1	(+) (+)	17.4	141.3	67.3	283.2
C2	(−) (−)	18.8	177.9	59.5	292.4
C3	(+) (−)	12.6	112.5	69.0	285.1
C4	(−) (+)	30.9	164.4	54.3	294.1

**Table 6:** Mass in kg of CO_2_ generated per kg of bioplastic at 30 days, after its biodegradation in farmland.

Code	Design	Mass of CO_2_ (kg)/kg of Bioplastic
C1	(+) (+)	0.2084 ± 0.0045
C2	(−) (−)	0.3847 ± 0.0102
C3	(+) (−)	0.2216 ± 0.0056
C4	(−) (+)	0.2688 ± 0.0063

**Table 7:** Analysis of variance for CO_2_ mass (kg)/kg of bioplastic films.

Source	MC Ajust.	F Value	*p* Value
A: *Aloe vera* concentration (% *w*/*w*)	0.037464	767.16	0.000 *
B: Eucalyptus oil concentration (% *w*/*w*)	0.012500	255.97	0.000 *
AB	0.007910	161.98	0.000 *

* Significance level (*p* ˂ 0.05).

## Data Availability

The data presented in this study are available on request from the corresponding author.
